# Experiment and Simulation Research on the Fatigue Wear of Aircraft Tire Tread Rubber

**DOI:** 10.3390/polym13071143

**Published:** 2021-04-02

**Authors:** Jian Wu, Long Chen, Da Chen, Youshan Wang, Benlong Su, Zhibo Cui

**Affiliations:** 1 Center for Rubber Composite Materials and Structures, Harbin Institute of Technology, Weihai 264209, China; chenl042574@163.com (L.C.); chendamec@163.com (D.C.); wangys@hit.edu.cn (Y.W.); rains853@163.com (B.S.); 2 School of Intelligent Manufacturing and Automobile, Chongqing Vocational College of Transportation, Chongqing 402260, China

**Keywords:** aircraft tire, fatigue wear, rubber, finite element model, pavement

## Abstract

The road surface and the tread pattern structures directly affect the wear performance of aircraft tire, especially for lateral sliding conditions. In this paper, wear tests of tread block with different draft angles and root radiuses, different interfaces, and different slip angles were carried out, and combined with the simulation, the effects of tread groove structure and slip angle on the wear mechanism were analyzed. Results indicated that the influences of draft angle were greater than the root radius; the wear geometry of the tread block decreased when the draft angle increased in the range of 0° to 15°, but for the root radius, the wear geometry of each sample was similar to a strip shape. A considerable material loss occurred at the front edge when the slip angle increased, and the slip angle was larger in the range of 0° to 45°. Combined with the simulation and wear test, fatigue wear and abrasive wear of the slide surface are dominant factors when considering the effects of tread groove structure and slip angle, and both front edges of the tread blocks roll up repeatedly; the coefficient decreases with the increase in load when the cement concrete pavement interface is dry, but for a wet interface, the coefficient decreases softly.

## 1. Introduction

The tire is the key component of an aircraft, and tread patterns are critical features for tire design as they also affect the wear performance, especially for lateral sliding and wet conditions [1,2,3]. Therefore, it is essential to study the influence of tread patterns and service condition on the wear resistance of aircraft tires.

Friction results in detachment of material particles from the contact surface. This process is generally denoted as abrasion or wear. The wear characteristics of rubber have attracted the widespread attention of researchers due to its destructive characteristic; the influences of temperature and speed on the rubber wear have been studied by Grosch and Schallamach [4]. The effects of surface texture and tread block geometry on the wear mechanism of tread rubber were analyzed. It was found that the micro texture of the pavement with good integrity had a significant impact on the durability of the tire tread rubber [5]. Cardoso [6] studied the wear resistance of tire tread rubber compounds by developing laboratory equipment. Further research into the friction and wear behavior was attained between rubber and steel plates. As a result, the rougher the counterpart surface, the more easily the friction pairs enter into the mixed lubrication regime, which is useful to reduce rubber wear and increase its lifespan [7]. Tread patterns directly affect the tribological and wear properties such as curling deformation of the front edge and change of the block contour (e.g., Capozza [8] and Gabriel [9]). Capozza indicated that macroscopic surface grooves were theoretically effective in reducing static friction; contrary to static friction, kinetic friction is weakly affected. Additionally, Gabriel discussed the influence of surface geometry on kinetic friction. From the experiments, the kinetic friction of a rubber slider is essentially affected by the number of grooves on the contact surface. The relation [10] between the frictional behavior and interface states is treated in detail: kinetic friction decreases as the number of grooves increases on the dry interface, but for a wet interface, the effect of the grooves on kinetic friction depends on the surface roughness of the rubber slider. A modified analytical model into the compression effects of tread block was presented in [11]. This paper showed that the narrow tread block was prone to curling deformation on the asphalt pavement. Furthermore, the effects of temperature and slip angle on the wear of aircraft tire tread materials were studied by Wu [12]. However, the structure optimization of tire performance, both indoor and outdoor tire tests, is enormously sophisticated, time-consuming, and costly [13]. Therefore, a consideration of single tread blocks or simplified tread patterns, cured based on the tread material, was the focus of this paper, which contributes to a deeper understanding of the wear behavior and the interactions between the tread patterns and pavement.

The aircraft tire is an important component. However, the periodic loads usually lead to fatigue cracks of the tire. Failure analysis and fatigue life prediction are also important for the safety and reliability of rubber components [14]. The finite element method (FEM) has become a convenient and powerful tool in mechanical engineering for product design and development, fatigue prediction, and heat generation, and can be completed by simulation when a new product is developed in the tire industry [15]. The effects of varied loads and air pressure on the tire wear characteristic were investigated with ABAQUS software [16,17]. The most crucial aspect of aircraft tire wear simulation is the realistic description of the processes in the contact interface between the tire’s tread and the road surface such as the imitation of the sliding condition, distribution of temperature, and stress of rubber block with different shapes [18,19]. In the work of Kongo [20], a non-uniform computed thermal flux was applied to the tire surface corresponding to the contact area and rotated around the tread to simulate the heat generation during the rolling process. Alroqi and Wang [21] succeeded in reducing the temperature rise by pre-spinning the wheel in their simulation. In [22], a thermo-mechanically model for the contact behavior of rubber and rough (road) surfaces was presented, where the sliding behavior of the tread blocks was considered, but the abrasion was not covered in the addressed model. Hofstetter [18] provided an extension of this model by a formulation for the determination of the abrasional material loss. 

The main emphasis in this paper addressed two topics. In the first part, wear tests of tread blocks with different draft angles and root radiuses were carried out and the effect of slip angle was investigated. Furthermore, the simulation of abrasion during sideship and the wear mechanism was analyzed by FEM. In the second part, the effects of asphalt pavement, cement, and concrete pavement on the friction characteristics were discussed, and the wet skid resistance between the tread rubber and cement and concrete pavement was studied.

## 2. Experimental Procedure

### 2.1. Materials

**Figure 1:**
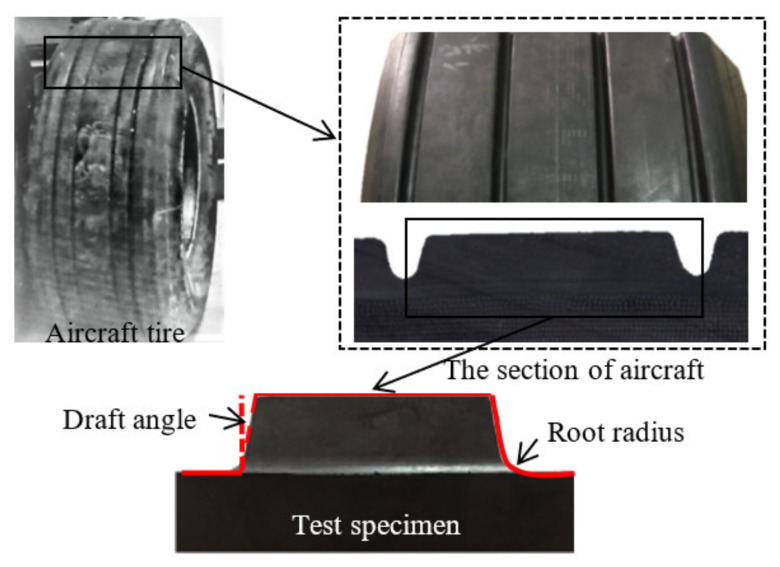
Geometry of the aircraft tire groove.

A simplified cross section of an aircraft tire is shown in Figure 1, where the tread block has complicated geometry, draft angles along the sides of the blocks, and root radiuses.

**Figure 2:**
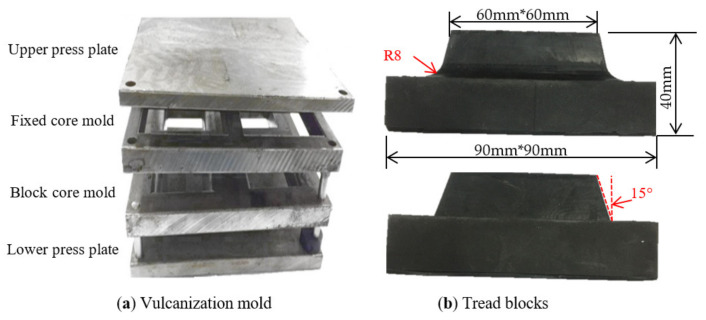
Vulcanizing mold and test samples.

Such tread blocks were cured by a vulcanization mold in a flat-panel press under 15 MPa, 160 °C for 35 min, as shown in Figure 2a. The vulcanization mold includes the upper and the lower press plates, block core mold and fixed core mold; the block core mold has different draft angles and root radiuses. Figure 2b shows a photo of a tread block as used in the wear test, and the main dimensions of the tread block are specified.

**Figure 3:**
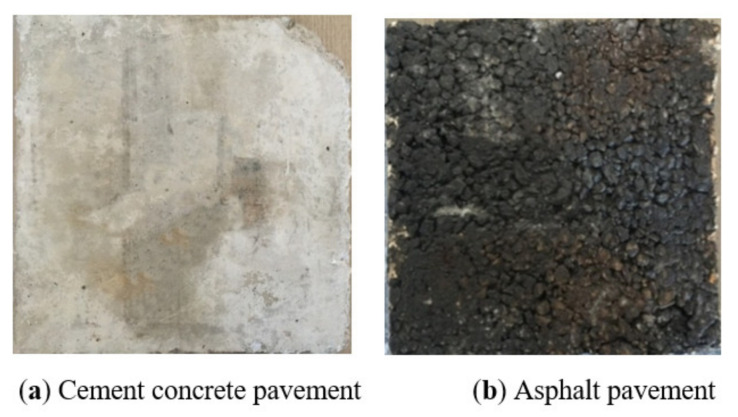
Pavements.

The pavement in our experiment was asphalt pavement and cement concrete pavement, as shown in Figure 3, and the replaceable pavement size was 300 mm × 300 mm × 40 mm [23]. The cement concrete pavement was performed according to the MH 5006-2015, and the asphalt pavement was performed according to MH/T 5010-2017, and were provided by the School of Transportation Science and Engineering at the Harbin Institute of Technology.

### 2.2. Experimental Method

**Figure 4:**
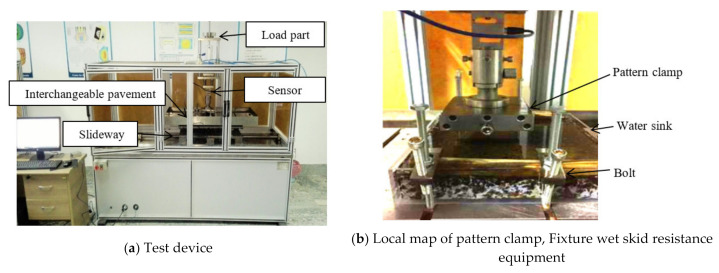
Wear test platform.

Figure 4a shows the tread rubber block test device [11], where the test device consists of a normal loading mechanism, horizontal feed mechanism, tangential force test facility and pattern clamp, etc. The normal loading mechanism is loaded with weights, and the load spindle can be adjusted in the range of 0°–90°. 

The test procedures can be described as follows: First, wear tests of different draft angles and root radiuses were carried out under the load of 300 N and the slip angle of 0°, and the velocity amounted to *v* = 60 mm/s. Furthermore, when the draft angle was 0° and the root radius was 0, the effects of slip angle were investigated. Table 1 shows the experimental parameters of the final tests; test samples were provided with reciprocating motion for 20 m (a reciprocating motion is 0.06 m); three tests were carried out under each condition. Then, the wear tests of the rubber blocks were carried out under different pavements and different loads, where the velocity amounted to *v* = 100 mm/min, the block and pavement were fixed by the pattern clamp and bolts. The wet skid resistance tests were performed by a developed test device, as shown in Figure 4b [23], where the water film depth was 1 mm, 0 mm represents the dry friction.

**Figure 5:**
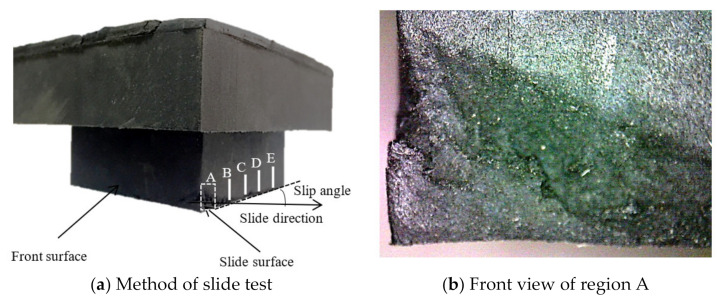
Test schematic of wear test.

A detailed discussion of the wear surface morphology at different regions was conducted by a DSX510 optical digital microscope such as regions A, B, C, D, and E. Figure 5 shows the test schematic of the wear test.

## 3. Finite Element Model

### 3.1. Material Constitutive Model

Material properties of tread rubber were characterized by the Yeoh model, which is given by Equation (1) [12]:

(1) $$\begin{array}{l} W=C_{10}(I_{1}-3)+C_{20}{\left(I_{1}-3\right)}^{2}+C_{30}{\left(I_{1}-3\right)}^{3}\\ \sigma =2[(1+\varepsilon )-{\left(1+\varepsilon \right)}^{-2}]\left\{\begin{array}{l} C_{10}+2C_{20}[{\left(1+\varepsilon \right)}^{2}+2{\left(1+\varepsilon \right)}^{-1}-3]\\ +3C_{30}{\left[{\left(1+\varepsilon \right)}^{2}+2{\left(1+\varepsilon \right)}^{-1}-3\right]}^{2} \end{array}\right\} \end{array}$$

where *W* is the strain density; *I*_1_ is the first invariant of the deviatoric strain tensor; and *C*_10_, *C*_20_, and *C*_30_ are the rubber material constants, which are obtained by the experimental work from the tread rubber formulation. Parameters are shown in Table 2 [12].

### 3.2. Meshing and Boundaries

**Figure 6:**
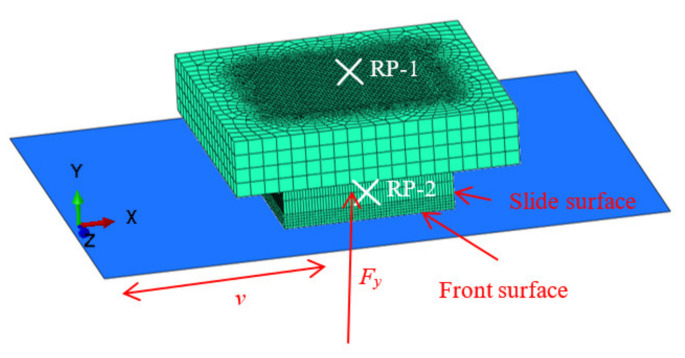
3D finite element model.

Figure 6 shows a 3D finite element model. The C3D8R element used for the tread block included 78,769 nodes and 64,368 elements. The reference node RP-1 of the tread block was fixed in the *y* and *z* direction displacement degrees of freedom (DOF) and all turn DOF, then a velocity of *v* = 60 mm/s was applied to the RP-1, and the total slide distance was 20 m throughout all simulations. The asphalt road was modeled as analytical rigid, the RP-2 of asphalt road was fixed in all displacement DOF, and *x*, *y*, and *z* turn DOF. 

### 3.3. Fatigue Wear Criterion

**Figure 7:**
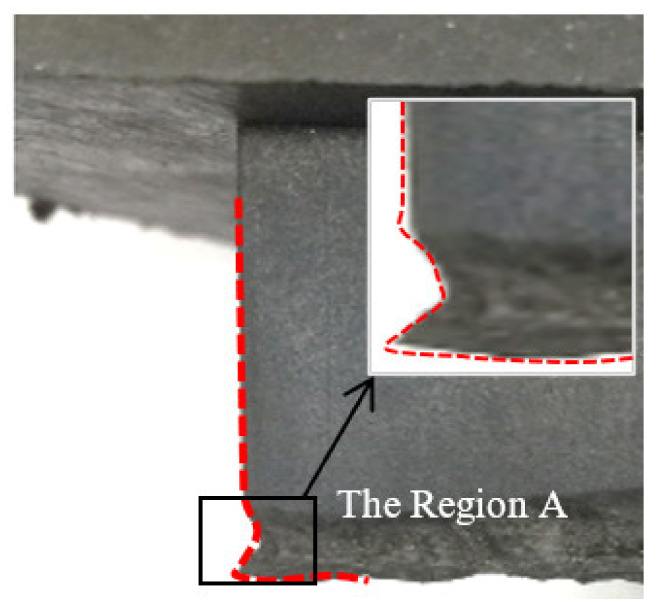
Wear loss of the region A.

Based on the wear tests with different conditions, the deformation behavior of each block was very similar [11]: both front edges rolled up, so that parts of the flanks made contact with the asphalt pavement; as shown in Figure 7. After reciprocating motion for 20 m, a considerable material loss occurred at the front edge, which was region A when the slip angle was 45°, which is depicted in detail. Here, material damage was taken into account in the simulations, and fatigue damages were adopted to characterize the wear mechanism. 

Strain and stress damage parameters are widely used to evaluate the fatigue life of rubber [24]. Maximum Green–Lagrange strain and maximum logarithmic strain are presented to research the effect of groove structure on fatigue wear. The maximum Green–Lagrange strain can be calculated by Equation (2) [25]:

(2) $$\varepsilon_{G}=\frac{{\left(\varepsilon_{E}+1\right)}^{2}-1}{2}$$

where *ε_G_* is the maximum Green–Lagrange strain, and ε*_E_* is the maximum logarithmic strain, which can be obtained directly by FEM.

## 4. Results and Discussion

### 4.1. Effect of Draft Angle

**Figure 8:**
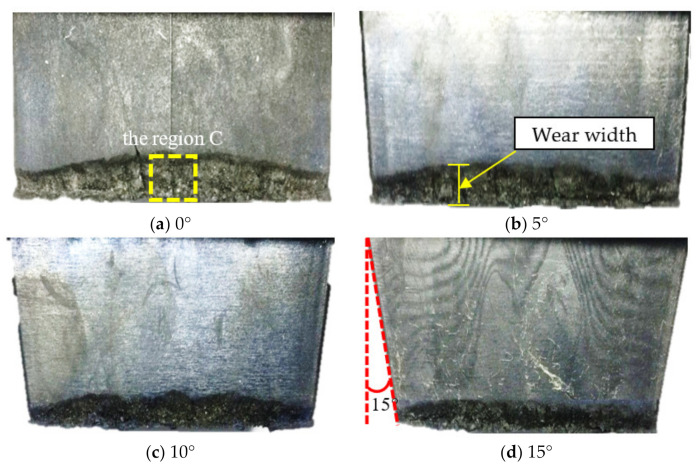
The changes in slide surface with draft angle.

**Figure 9:**
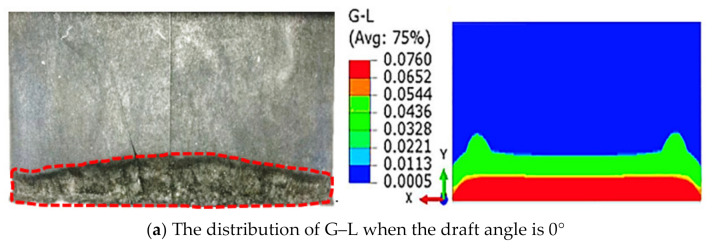

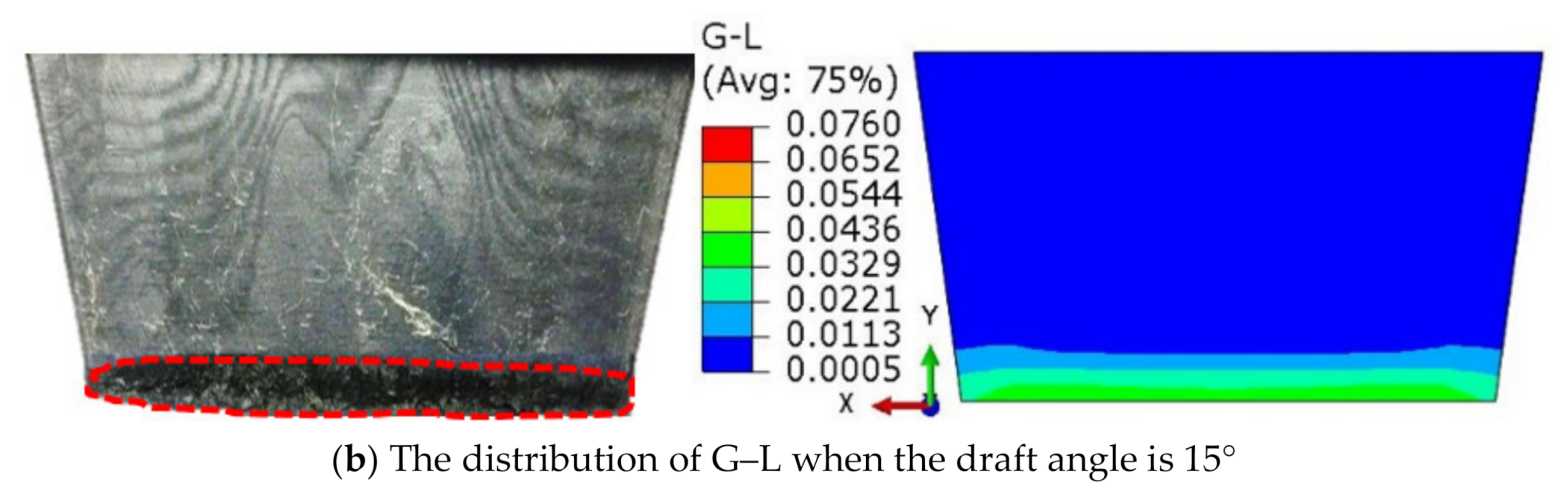
The simulation results of G–L and into the effects of slip angle.

Draft angle is important for the design of an aircraft tire, and determines the safety of the aircraft. Wear mainly occurs on the slide surface, which is the same as the speed forward direction. Figure 9 shows the effects of draft angle on the slide surface. It can be seen that draft angle has a significant impact on the generation and growth of the wear scar, where the wear of each block is very similar, and both blocks had severe wear at region C, as shown in Figure 8a, and wear decreases along the bottom side. Wear of the same region decreased with an increase in draft angle. Here, the wear width of the front view are discussed, as shown in Figure 8b.

Combined with the simulation results of G–L, as shown in Figure 9, with the increase in draft angle, the distributions of the G–L strain was in good agreement with the test results. Damage parameters can be used to evaluate the fatigue wear.

**Figure 10:**
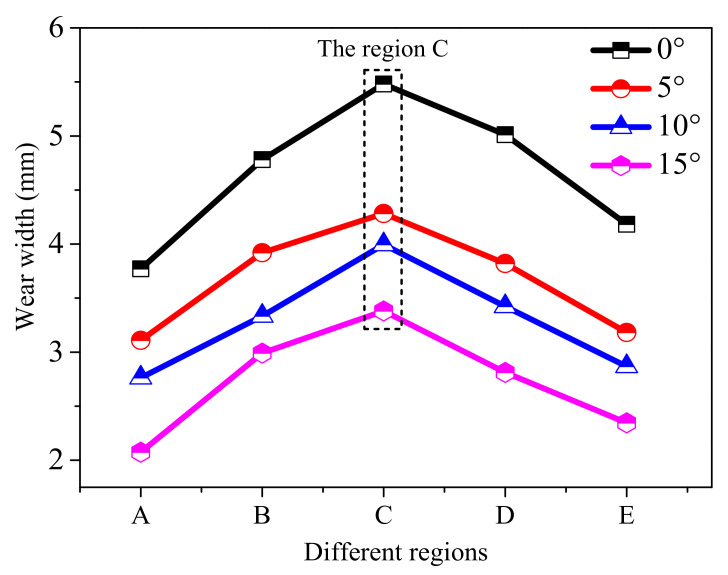
The changes of wear width with draft angle and wear region.

Further analysis of different regions on wear width was carried out, as shown in Figure 10. Wear width of the same region decreased with an increase in draft angle such as in region C. 

### 4.2. Effect of Root Radius

**Figure 11:**
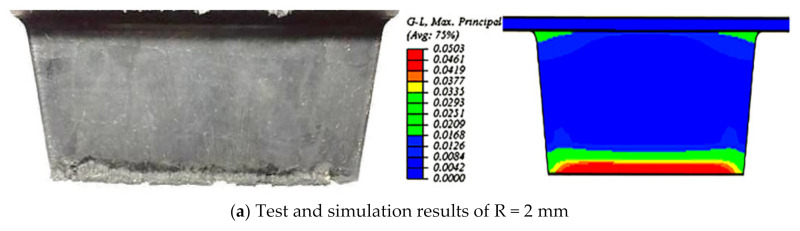

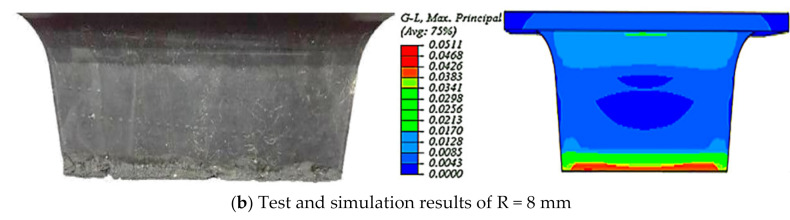
Comparison of the test and simulation results of the slide surface.

As seen in Figure 11, the results showed the qualitative agreement of the extent of the contour change under different root radiuses, and the shape of the wear scar on the slide surface was similar to a strip shape because the damage parameter G–L almost remained a constant value with variation of root radius, which resulted in the same deformation under different root radius.

### 4.3. Effect of Slip Angle

**Figure 12:**
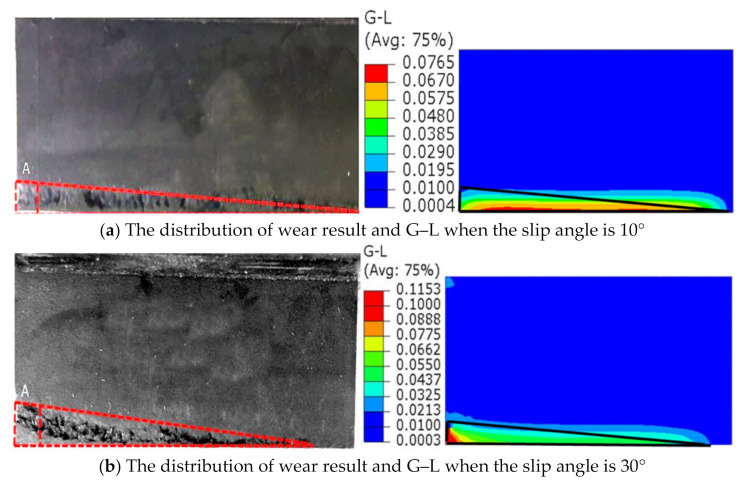
The changes of the slide surface with slip angle.

When the draft angle was 0°, the root radius was R0, and the additional effects of slip angle were studied. Figure 12 shows the wear surface considering the effect of slip angle. 

Wear was still observed on the slide and front surface, which was different from the wear distribution of the draft angle. The worst wear occurred at the junction of the slide surface and front surface, as seen in region A, where reciprocating motion resulted in a high peak of G–L at region A, as shown in Figure 12b. Wear decreased along the bottom side of the slide surface because the max Green–Lagrange strain occurred at the front edge, and distributions of the Green–Lagrange strain were very similar to the wear tests. When the slip angle increased, the zone of higher G–L moved toward the junction of the slide surface and front surface and mechanical performance degradation happened immediately, abrasion became inevitable, which showed good consistency with the experiment.

**Figure 13:**
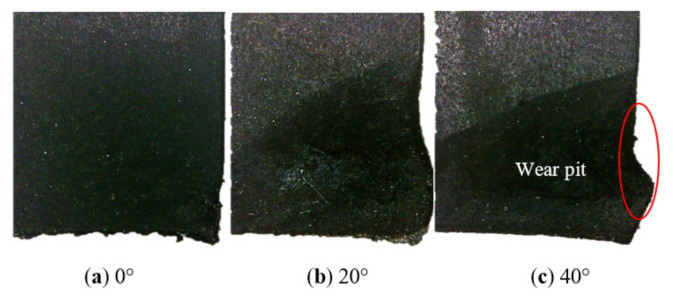
The changes of the junction of the slide surface and front surface with slip angle.

Here, with the slip angle increased, the pits also increased, as shown in Figure 13. In this condition of round-trip wear, it was seen that the tread block with a large slip angle could be worn off first.

**Figure 14:**
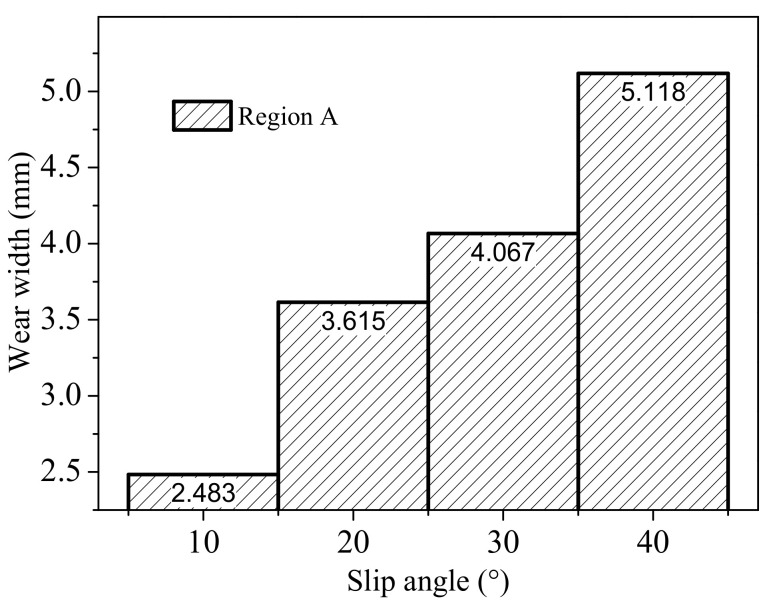
The variation of wear width with slip angle and wear region.

Figure 14 shows the wear width of region A. Results indicate that with the increase in slip angle, the average wear width of different region increases as the larger slip angle further boosts the local (unrecoverable) deformations of the rubber block at the slide edge.

### 4.4. Effects of Pavements

**Figure 15:**
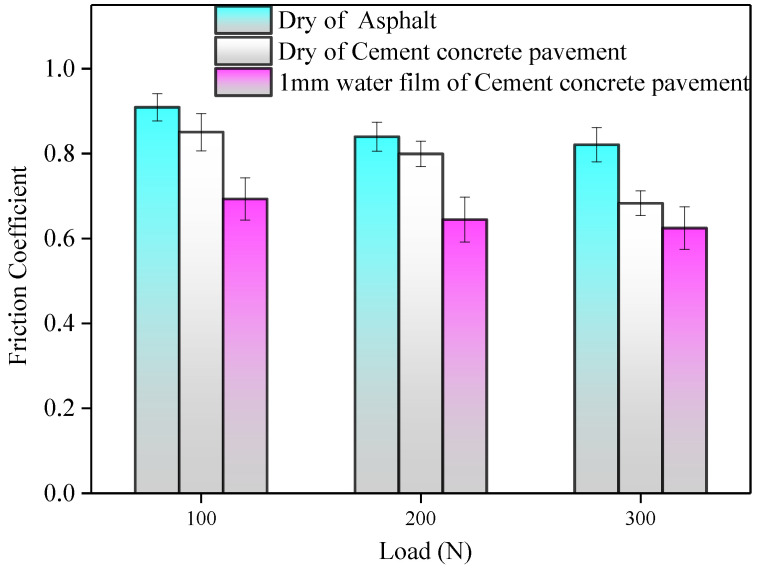
The variation of coefficient with load.

Figure 15 shows the friction coefficient variation of different road surfaces with a speed of 100 mm/min. It can be seen that the coefficient of the asphalt pavement was significantly greater than that of the concrete pavement under the same load. Compared to the dry friction, the contact forms between the tread rubber block surface and cement pavement cannot be broken completely in shallow water film as the water film can be squeezed into fully thin when the tread rubber block slides at a certain speed. Therefore, the friction coefficient decreases when the normal load increases [23].

**Figure 16:**
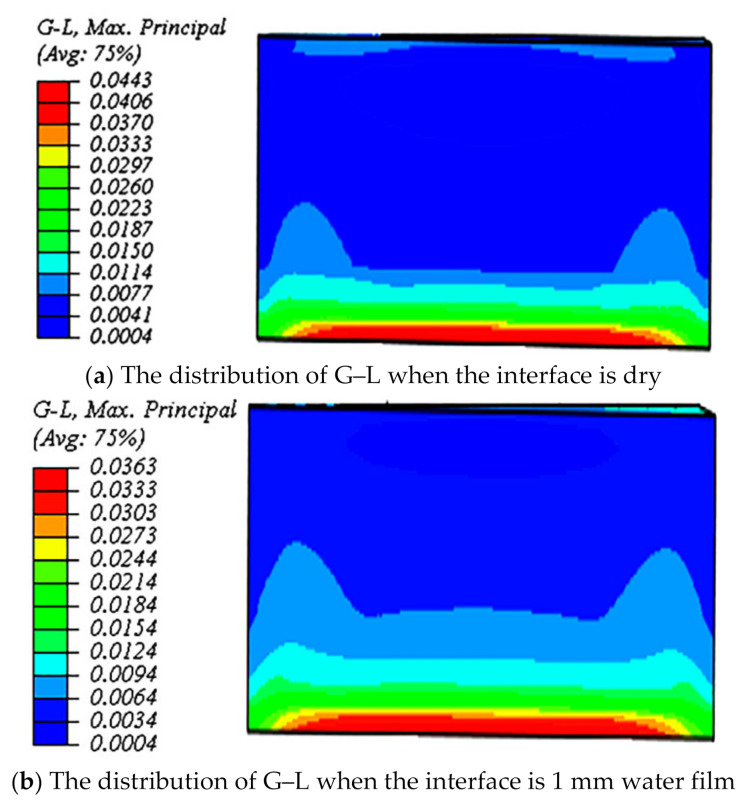
The simulation results of the G–L considering the effects of interface condition.

Combined with the tests, the simulation results indicate that under the same condition, the G–L of the dry condition was larger than that of the 1 mm water film, and the wear of the dry concrete pavement was more severe. Figure 16 shows the distribution of the G–L.

## 5. Conclusions

The effects of the groove structures, wet condition, and contact pavement on the wear characteristics of the tread block considered were studied. Furthermore, the effect of slip angle on tread block wear have been discussed in detail. According to the results and the analyses above, the main conclusions are given as follows:

(1) With the increase in the draft angle, the wear of tread block at the same region continuously decreased; root radius had little impact on the wear geometry, and the shape of the wear scar of the slide surface was similar to a strip shape. Fatigue wear and abrasive wear of the slide surface are dominant factors when considering the effects of tread groove structure and slip angle.
(2) The effect of slip angle was different with the draft angle and the root radius. The worst wear was investigated at the junction of the slide surface and front surface, then decreased along the slide surface.
(3) The coefficient of the asphalt pavement was greater than that of the concrete pavement when the speed was 100 mm/min; the coefficient decreased with the increase in load when the interface was dry, but for a wet interface, the coefficient decreased softly.

## Figures and Tables

**Table 1:** Experimental conditions of tread rubber wear.

Influence Factors	Testing Conditions
Velocity (mm/min)	60
Sliding total distance (m)	20
Draft angle (°)	0,5,10,15
Root radius (mm)	2,5,8,11
Slip angle (°)	0,10,20,30,40,45

**Table 2:** Material constants [12].

Parameters	*C*_10_	*C*_20_	*C*_30_
	0.38	−4.46	7.03

## Data Availability

The data presented in this study are available on request from the corresponding author.
